# Placental Ageing in Adverse Pregnancy Outcomes: Telomere Shortening, Cell Senescence, and Mitochondrial Dysfunction

**DOI:** 10.1155/2019/3095383

**Published:** 2019-05-22

**Authors:** Samprikta Manna, Cathal McCarthy, Fergus P. McCarthy

**Affiliations:** ^1^Irish Centre for Fetal and Neonatal Translational Research (INFANT), Cork University Maternity Hospital, Cork, Ireland; ^2^Department of Pharmacology and Therapeutics, University College Cork, Western Gateway Building, Cork, Ireland

## Abstract

Preeclampsia is a multisystemic pregnancy disorder and a major cause of maternal and neonatal morbidity and mortality worldwide. The exact pathophysiology of preeclampsia remains unclear; however, it is speculated that the various pathologies can be attributed to impaired vascular remodelling and elevated oxidative stress within the placenta. Oxidative stress plays a key role in cell ageing, and the persistent presence of elevated oxidative stress precipitates cellular senescence and mitochondrial dysfunction, resulting in premature ageing of the placenta. Premature ageing of the placenta is associated with placental insufficiency, which reduces the functional capacity of this critical organ and leads to abnormal pregnancy outcomes. The changes brought about by oxidative insults are irreversible and often lead to deleterious modifications in macromolecules such as lipids and proteins, DNA mutations, and alteration of mitochondrial functioning and dynamics. In this review, we have summarized the current knowledge of placental ageing in the aetiology of adverse pregnancy outcomes and discussed the hallmarks of ageing which could be potential markers for preeclampsia and fetal growth restriction.

## 1. Introduction

Preeclampsia can be defined as de novo hypertension after 20 weeks of gestation in the presence of proteinuria and maternal organ/uteroplacental dysfunction [1]. It is one of the leading causes of maternal and neonatal morbidity and mortality affecting about 3 to 8% of pregnancies worldwide [2]. Preterm birth as a result of iatrogenic delivery is a common factor in preeclampsia with up to 25% of babies born to preeclamptic mothers being growth restricted [2, 3]. Preeclampsia and IUGR are pregnancy-specific disorders that are associated with placental insufficiency [4]. Complications associated with preeclampsia are responsible for about 15% of maternal deaths and high perinatal mortality rates worldwide [5]. Ageing is experienced inevitably by organs; however, in preeclampsia, the presence of persistent oxidative stress accelerates this process resulting in premature ageing of the placenta [6]. It has been reported by various studies that premature placental ageing is associated with telomere shortening, cellular senescence, and mitochondrial dysfunction [7–10].

## 2. Physiology of Ageing

Ageing is a unidirectional phenomenon experienced inevitably by every tissue. It can be characterised as cellular senescence through decline in functionality and mitochondrial dysfunction through altered metabolism and signalling.

### 2.1. Cellular Senescence

Cells are capable of replication, dividing into two exact copies of the parent cell. This includes division of the genetic material by unwinding and splitting of the DNA strands, in order to form two new copies as daughter strands. Telomeres are cap-like nucleotide repeats (TTAGGG) present on the end of each strand. Damage to DNA strands activates signalling cascades (ataxia telangiectasia-mutated (ATM) and ataxia telangiectasia and Rad3-related (ATR) pathways) that arrest cell division, induce cellular senescence, and promote cell death [11]. Telomeres with other proteins like shelterin protect the end of nuclei from being recognised as a double-strand damaged region (DDR) [11]. It has been shown that telomeres shorten after every replication cycle [12]. With the increase in proliferation and decrease in telomere size, cells enter senescence or a growth retardation phase (Hayflick limit) which ultimately results in cellular death [12]. Telomere shortening has been associated with various genetic diseases such as progeria or premature ageing and Hutchinson-Gilford syndrome [13].

In some cases, such as cancer, cells can bypass senescence and keep proliferating, becoming immortal. However, even cancer cells do not divide indefinitely, and they enter a crisis stage which results in either cellular death or telomere length stabilization with a consequent upregulation of the telomerase enzyme to maintain telomere length [14]. Telomerase is a RNA-protein complex that replenishes the lost nucleotides to preserve the length of the telomere [15]. In somatic cells, telomerases are turned off as a protective mechanism to prevent neoplastic changes [16], but their activity is more pronounced in germ cells, stem cells, and 90% of cancer cells [17].

### 2.2. Mitochondrial Dysfunction

**Figure 1:**
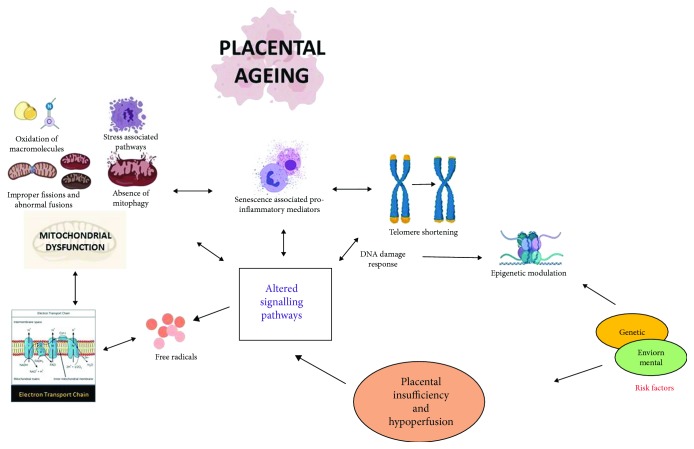
Cause and consequence of placental ageing: the pathophysiology of placental conditions such as preeclampsia and fetal growth restriction is often associated with internal and external factors such as oxidative stress, genetic, immunological, and environmental. This can result in placental insufficiency with further evidence of premature ageing of the placenta as a consequence of cellular senescence and mitochondrial dysfunction [130, 131].

Mitochondria are key regulators of metabolism, redox balance, and apoptosis. During the electron transport chain (ETC), electrons are pumped across the inner mitochondrial membrane (from reduced compounds to molecular oxygen) to create an efflux for synthesis of energy [18]. According to the free radical or oxidative stress hypothesis of ageing, there are a number of timeline events occurring during oxidative phosphorylation resulting in leaching of electrons from mitochondrial membranes which reacts with oxygen, forming free radicals such as superoxide, ultimately impairing redox balance [19, 20]. The free radical theory has been succeeded by the damage theory hypothesis which suggests that ageing can be associated with the frequent and inevitable cumulative damage at a molecular level caused by accumulated reactive oxygen species (ROS) and its by-products and enzymes [21]. Accumulated reactive oxygen species (ROS) leads to reversible and irreversible changes within cells, resulting in loss of molecular functioning and increased oxidative stress, a phenomenon frequently associated with senescence, as shown in Figure 1 [19, 20].

Mitochondrial functioning in ageing has been studied extensively; it has been reported by a number of articles that mitochondrial density, respiratory capacity, and ATP production decline with cellular ageing [22–24]. Apart from reduced functionality, ageing is accompanied with damaged mitochondrial DNA (mtDNA), mutations, and mitophagy [22, 25].

Impaired redox signalling induces various modifications within the macromolecules and mutations within mtDNA; however, deleterious effects of these mtDNA mutations are still not fully elucidated [26–28]. The bottleneck effect, which explains genetic drifts and variations in small population subsets, as is the case with mtDNA mutations, may transmit small variations in germlines which are amplified in later stages of oogenesis [29, 30]. A few studies have reported that mutations in yeast mtDNA result in a shorter lifespan when mitophagy is inhibited [31, 32]. Reduced mtDNA copy numbers have been associated with ageing and associated diseases including cardiovascular disease (cardiomyopathy, atherosclerosis), neurodegenerative disease (Alzheimer's disease, Parkinson's disease), cancer, and premature ageing of ovaries in women [33, 34].

Mitophagy is a protective mechanism to maintain mitochondrial quality, remove mutated mtDNA, and ensure mitochondrial homeostasis [35]. Furthermore, mitochondrial dynamics are maintained through frequent mitochondrial fission and fusion [36]. Oxidative damage to the mitochondria can cause impaired fission with asymmetric membrane potentials of daughter mitochondria which fail to fuse effectively, resulting in accumulated malformed mitochondrial proteins with decreased autophagic capacity [37]. Palikaras et al. showed that mitophagy is required for longevity in *C. elegans* as a result of reduced insulin/IGF-1 signalling or impaired mitochondrial function [38].

Furthermore, loss of mitochondrial biogenesis through an imbalance of mitophagy and mitochondrial fission/fusion and accumulation of mitochondrial debris leads to cardiac ageing in mice [39, 40].

Impaired mitochondrial functioning is reflective of the mitochondrial unfolded protein response (UPR^mt^) [41] which is regulated by communication between mitochondrial and nuclear proteins, where nuclear remodelling is required for the activation of UPR^mt^ [42]. When stress is induced within cells, UPR^mt^ is upregulated to promote repairs, but in cases of prolonged UPR^mt^ action, mutations may be induced within the mtDNA [42]. Experiments on *C. elegans* have shown that UPR^mt^ upregulation and imbalance in mitonuclear response induce longevity [43, 44].

Cell senescence is a result of nuclear damage usually associated with high oxidative stress and accumulation of ROS, debris, or misfolded proteins. Innate immune mediators with Senescence-Associated Secretory Phenotypes (SASPs) including cytokines, chemokines, growth factors, and proteases are responsible for clearing debris from dead or dying cells which might activate proapoptotic p53 and AKT signalling pathways [45–47]. In several studies, removal of inflammatory mediators and consequent signalling caused a reversal in cellular ageing, indicating that inflammation might be both cause and consequence in cellular senescence [48–50].

## 3. Pathophysiology of Preeclampsia

The pathophysiology of preeclampsia, even after decades of research, is not completely understood. Preeclampsia can be characterised as early onset, comprising or less than 20% of all cases, or late onset, comprising the remaining 80% [51]. Several hypotheses have been proposed to fully elucidate the underlying pathological mechanisms [51–54]. The placental origin of preeclampsia hypothesis describes inefficient trophoblast invasion and remodelling of maternal spiral arteries, causing “placental syndrome,” subsequently resulting in preeclampsia and fetal growth restriction [51, 54].

A more recent theory proposed by Thilaganathan implicated abnormal cardiovascular function driving the abnormal placentation that occurs within preeclampsia, and the placental origin of preeclampsia only holds true in the case of early onset of the disease [52, 54]. Late-onset preeclampsia can be attributed to underlying maternal cardiovascular dysfunction which fails to meet the haemodynamic and metabolic needs of the pregnancy and also results in adverse postpartum cardiovascular outcomes in over 50% of patients, possibly due to the fact that both preeclampsia and cardiovascular diseases share similar genetic and environmental risk factors [52].

Supporting the link between placental-mediated pathophysiology of early-onset PE, Yung et al. examined placental samples from preterm, term, and second trimester pregnancies and demonstrated that in early-onset preeclampsia, placental pathology is associated with upregulation of the unfolded protein response (UPR) pathway and ER stress activation along with depression of the AKT pathway, reducing cellular proliferation [55]. These authors demonstrated molecular differences between the two phenotypes of term and preterm preeclampsia [55].

Roberts and Hubel, amongst others, proposed a two-stage model of preeclampsia which was later modified to six stages by Redman et al. [53, 56]. Redman explains that stages 1 and 2 could be an early phenomenon such as preconception tolerance of the mother towards paternal semen or short duration between coitus and conception, leading to poor placentation and affecting the health and growth of the fetus [53]. Stage 3 begins at around 8 weeks of pregnancy where in uncomplicated cases, trophoblastic plugs sealing the maternal spiral arteries recede and the establishment of uteroplacental circulation begins [53, 57]. However, in preeclampsia, there is premature opening of trophoblastic plugs with the influx of arterial blood increasing stress within the placenta and leading to defective placentation [53]. Stage 4 of Redman's model is characterised by decrease in placental growth factors in cases of preeclampsia, leading to impaired vascular remodelling of maternal spiral arteries into larger vessels with low resistance to blood flow by trophoblast [53, 58, 59]. Furthermore, Redman also states that with increased placental and endothelial damage, clinical symptoms of preeclampsia are evident in stages 5 and 6 [53].

The development of the placenta begins with a decidual reaction evoked by the blastocyst in the maternal endometrium, eventually leading to the formation of the basal plate [60, 61]. Trophoblasts differentiate into two types: the invasive extravillous cytotrophoblast (EVT) or the villous cytotrophoblast which later fuse to form the multinucleated syncytiotrophoblast [61, 62]. The extravillous cytotrophoblasts are responsible for establishment of uteroplacental circulation and regulation of the maternal innate immune system [63, 64]. In uncomplicated pregnancy, extravillous cytotrophoblasts (EVT) invade maternal spiral arteries, veins, and lymphatic vessels of the endometrium during the first trimester [65, 66]. EVT accumulations have also localised in lymph nodes without any neoplastic changes suggesting immune cell regulation by trophoblasts [65, 66].

Defective placentation results in a decrease in the angiogenic vascular endothelial growth factor (VEGF) and placental growth factor (PLGF) and the release of deleterious placental factors like soluble fms-like tyrosine kinase 1 (sFIt1) into the maternal circulation causing generalized endothelial dysfunction [56, 57]. Circulating sFlt1 is abundant in preeclamptic women which is attributed to vascular resistance and increase in arterial blood pressure [67]. Recent studies in women with fetal growth restriction (FGR) with no symptoms of preeclampsia have provided evidence of a high sFlt1/PLGF ratio [68, 69]. Proteinuria in preeclampsia can also be associated with high levels of placental factors like sFlt1, precipitating glomerular endotheliosis [70]. Altered haemodynamic and decreased vascular flow leads to persistent hypoxic-oxygenated states, resulting in high oxidative stress within the placental tissue [55, 71–74]. Even though elevated oxidative stress is evident in trophoblasts during uncomplicated pregnancy, prolonged oxidative stress suppresses trophoblast activity, worsening the situation in pregnancy complications [75].

The syncytiotrophoblast is the outermost layer of the villous trophoblast, in direct contact with maternal blood, and syncytial debris in the form of knots or nuclear aggregates can be detected in maternal circulation with increasing gestational age [76, 77]. Constant vascular flow to the syncytiotrophoblast causes rapid ageing changes and requires activation of autophagy for their removal in uncomplicated pregnancies [8, 78, 79]. During preeclampsia, the syncytiotrophoblast experiences accelerated ageing with upregulation of the apoptotic cascade, necrotic breakdown with release of necrotic debris, and increase in syncytial aggregates [77, 80]. It has been observed that the syncytiotrophoblast secretes Senescence-Associated Beta-Galactosidase (SA*β*-Gal) along with elevation of proapoptotic p53 and CDK inhibitors, indicating cessation of cell cycle and senescence [81, 82].

A recent review by Cox and Redman outlines the biochemical pathway and the mechanism of ageing of placental cells following oxidative stress [8]. They outline that the early onset of senescence in preeclampsia can be due to excessive ROS accumulation and oxidative/endoplasmic reticulum (ER) stress leading to mTORC pathway (cell cycle regulator) activation and production of Senescence-Associated Secretory Phenotype (SASP) proteins which then activates the cyclooxygenase pathway and enhances generation of proinflammatory cytokines and chemokines [8].

## 4. Cellular Senescence in Adverse Pregnancy Outcomes

Trophoblasts mimic cancer cells by displaying invasiveness in order to support growth of the fetus. Like neoplastic cells, trophoblasts maintain their telomere length and Human Telomerase Reverse Transcriptase (hTERT) level in uncomplicated pregnancies [83, 84]. During the first trimester, the trophoblast experiences low oxygen tension or a physiologic hypoxic state, associated with the upregulation of HIF-1*α*. This low oxygen tension state is responsible for the modelling of villous architecture and maintenance of cellular integrity by preservation of telomere length and upregulation of telomerase enzymes [85, 86]. This physiologic state is altered at the end of the first trimester when the placenta becomes oxygenated [87]. The telomere length remains constant throughout normal pregnancy, but in certain conditions like fetal growth restriction and uncontrolled diabetes, telomere length is significantly reduced [88, 89]. Telomere changes could be associated with increased oxidative stress, leading to DNA damage and activation of damage response (DDR) through the p53 pathway and promoting senescence of the trophoblast [9, 84]. In 2010, Biron-Shental et al. were the first to report evidence of cellular senescence in preeclampsia and fetal growth restriction. They demonstrated that in preeclampsia and IUGR pregnancies, telomeres are significantly shorter in the trophoblast with reduced expression of hTERTs with increased frequency of telomere aggregates when compared to uncomplicated pregnancies [90]. These placental alterations were not present at 37-41 weeks' gestation, indicating premature ageing of the placenta in these pregnancy complications [90].

The placenta ages gradually, and the presence of senescence markers such as p21, p16, p53, and Rb proteins towards the term supports the ageing hypothesis of the normal placenta [91]. Recently, a study by Nuzzo et al. shows cell cycle changes with upregulated cyclin D1 (cell cycle regulator) and PARP1 (expressed on DNA damage and age-related changes) with downregulation of JunB (senescence suppressor gene) in placental mesenchymal stromal cells (PMSC) extracted from the preeclamptic placenta when compared to the normal placenta [92]. Additionally, Sharp et al. found an imbalance between p53 (senescence-inducing and proapoptotic gene) and MDM2 (p53 suppressor) signalling in placental proteins in the syncytiotrophoblast but not in the cytotrophoblast in patients with early-onset preeclampsia [93]. Finally, Gao et al. reported the upregulation of p53 signalling and cell cycle arrest with activation of proapoptotic BAX and caspase proteins in human umbilical vein endothelial cells (HUVEC) taken from patients with preeclampsia when compared to that from uncomplicated pregnancy controls [94].

Activation of DDR pathways, mtDNA mutations, and endogenous stress like ROS accumulation and redox signalling can cause epigenetic modifications. Epigenetics can be defined as physiologic changes which cannot be attributed to the genetic code but rather modifications like phosphorylation, acetylation, or methylation of DNA or histone proteins in the nucleus [95]. Either these changes could be an adaptive response to persistent stimuli or they could be virulent modifications leading to irreversible alterations in germ cells [96]. The link between epigenetics, ageing, and age-related disorders has been demonstrated in several studies. Remodelling of chromatin may include methylation or demethylation of histone residues at specific sites, which can induce epigenetic changes within the cells [95, 96]. Lowe et al., through their research on endothelial cells collected from the coronary artery of a 19-year-old male, discussed that cellular ageing can be estimated precisely through intrinsic properties of cells known as the epigenetic clock indicated by specific DNA methylation or cytosine methylation of CpG islands, which may speed up ageing under certain conditions [97]. Madrigano et al., through their longitudinal study in an elderly population, showed that gene-specific DNA methylation is strongly associated with ageing and age-related disorders [98].

Epigenetic changes have been demonstrated within placental tissue including DNA methylation patterns, binding ability of DNA-binding proteins or DBP (especially in the cytochrome p450 gene), methylation patterns on imprinted regions in H19/IGF2 genes, and methylation of histone proteins within the human growth hormone in placental chromatin [99]. In a recent study by Eddy et al., histone modifications were evident in the BeWo placental trophoblast when exposed to hypoxic conditions with marked hypomethylation of cytosine, an epigenetic change usually evident in age-related pathologies [100].

## 5. Mitochondrial Dysfunction-Associated Ageing Changes in the Placenta

The placenta is a highly metabolic organ, requiring robust mitochondrial activity. Even with normal mitochondrial function, continuous changes within the extravillous trophoblast (EVT) and vascular remodelling lead to increased ATP production through oxidative phosphorylation [101, 102].

Accumulation of ROS such as superoxide, nitric oxides, and peroxynitrite results in oxidative stress during early pregnancy [101]. ROS are signalling molecules which have predominance over apoptotic, redox, and inflammatory-mediated signalling pathways, respectively [103, 104]. ROS can be generated from a number of sources including mitochondria (mROS), NADPH oxidases, xanthine oxidase, or p450 enzyme [105, 106]. Reactive oxygen species (ROS) alter macromolecules through oxidative damage to proteins affecting gene functioning, lipid peroxidation with increased polyunsaturated fatty acid circulation, and loss of cellular membrane integrity [107–109]. Upregulation of ROS and its by-product generation, lack of clearance, and redox signalling have direct links to both mitochondrial and cellular ageing. In addition to these changes, oxidative stress activates the Mitogen-Activated Protein Kinase (MAPK) pathway via the Stress-Activated Protein Kinase/Jun N-Terminal Kinase (SAPK/JNK) or p38 pathway in response to DNA damage, which further leads to cessation of cell proliferation, and cellular senescence [110].

Within the placenta, protein nitration and lipid peroxidation have been shown to be significantly higher in tissue isolated from preeclampsia patients compared with uncomplicated pregnancy [111–113]. Lipid peroxidation can be either enzymatic, catalysed by lipoxygenase enzymes, or nonenzymatic in the presence of free radicals [114]. Nonenzymatic lipid peroxidation is upregulated with ageing, and its products are highly expressed in age-related diseases such as atherosclerosis [115]. In 1998, Wang and Walsh described that placental lipid peroxidation was significantly higher in preeclampsia [111]. They established that high lipid peroxidation levels are a result of escalated mitochondrial mass in poor vascular flow to meet energy requirements in preeclampsia [111].

Various systematic reviews have indicated elevated lipid oxidation levels in preeclamptic maternal serum and plasma, usually associated with impaired antioxidant defence when compared with uncomplicated pregnancies [116, 117].

Low vascularization of the placenta results in a reduction in mitochondrial size, number, and activity [118, 119]. A recent study by Venkata et al., using the reduced uterine perfusion pressure (RUPP) model of preeclampsia in rats, showed significant reduction in mitochondrial respiration, especially in stage 3, and increased hydrogen peroxide production when compared to control rats [118].

Nitric oxide signalling regulates cell processes such as apoptosis, differentiation, and division [120]. Extravillous trophoblastic activity such as migration and invasion could be affected by two subtypes of nitric oxides, eNOS (endothelial nitric oxide synthase) and iNOS (inducible nitric oxide synthase) [120]. Nitration of proteins may either have no effect or exhibit loss/gain of functionality. In the placenta, peroxynitrite may alter vascular reactivity and uteroplacental circulation in pregnancy complications such as preeclampsia [91, 121]. Upregulation of NOS activity and peroxynitrite accumulation in preeclampsia may result in depression of mitochondrial activity by inhibiting the electron transport chain and nitration of mitochondrial proteins and antioxidants such as mitochondrial superoxide dismutase (MnSOD) along with activation of trophoblastic apoptosis [122].

As shown in Figure 1, ageing is usually associated with mitochondrial dysfunction as a direct consequence of loss of mitochondrial dynamics and increased mitochondrial damage [122, 123]. Mitochondria undergo continuous fission and fusion to maintain mitochondrial dynamics, failure of which may lead to accumulation of mitochondrial debris and deteriorate mitochondrial functioning [122, 123]. It is believed that mitochondrial dynamics are altered in preeclampsia with altered expression of mitochondrial autophagy regulator genes like OPA1, SIRT3, and MNF2/1, but limited research has been conducted to fully understand the signalling process [124, 125]. Recently, Ausman et al. reported that mitochondrial dynamics in preeclampsia is inclined towards mitochondrial fission with increased phosphorylation of the DERP1 gene in mitochondria isolated from preeclamptic placentas [126]. These changes were accompanied by accumulation of ceramides which upregulated BOK, a proapoptotic protein in preeclamptic placental tissues when compared with normal placenta indicating increased trophoblastic autophagy in preeclampsia and IUGR [126].

Antioxidants are produced by cells to neutralise ROS products by converting them to oxygen and hydrogen peroxide, which is further broken down to form water. Antioxidants are also considered as antiageing agents and can be classified into subtypes: enzymatic such as superoxide dismutases (SODs), catalases, and peroxidases or nonenzymatic such as vitamins (A, C, and E), bilirubin, and uric acid [127, 128]. Oxidative stress ensues when there is an imbalance between the ROS by-products produced and those scavenged by antioxidants [128, 129]. All major antioxidants are localised in the placenta to reduce oxidative stress, and their activity is dependent on the amount of stress experienced by the cells [71]. In preeclampsia and IUGR, antioxidant activity is attenuated when compared to uncomplicated pregnancies, as shown in Figure 1 [127].

## 6. Senescence Markers in Adverse Pregnancy Outcomes

A major obstetric challenge has been to establish biomarkers for detection of pregnancy complications such as preeclampsia and IUGR in maternal body fluids. Various markers of mitochondrial dysfunction and oxidative stress have been investigated as early biomarkers for pregnancy disorders, as shown in Table 1. In 2012, Qiu et al. were the first to report elevated mtDNA levels in the maternal peripheral blood in preeclampsia indicating high oxidative stress and mitochondrial dysfunction [132]. A recent case-control study by Williamson and McCarthy and Kenny found elevated mtDNA copy numbers in maternal plasma at 15-20 weeks' gestation in preeclamptic cases compared to those in uncomplicated pregnancy controls [133, 134]. Furthermore, this study also demonstrated significant reduction in the antioxidant mitochondrial superoxide dismutase (mSOD) level at 15 weeks' gestation in women who later developed preeclampsia, thus vindicating mitochondrial dysfunction as a pathophysiological event in preeclampsia [134]. Another similar study by Marschalek et al. indicated similar results with elevated mtDNA copy numbers in maternal serum detectable in early-onset preeclampsia [135].

Apart from mitochondrial dysfunction, inflammation and inflammatory mediators are evident in premature ageing of the placenta. Certain ageing-associated inflammatory markers such as Senescence-Associated Secretory Phenotypes (SASPs) are elevated in preeclampsia [136].

Kupferminc et al. showed that TNF-*α* is significantly elevated in amniotic fluid and maternal plasma in severe preeclampsia [137]. sST2 (soluble ST2) is a member of the interleukin-1 family, expressed abundantly in endothelial cells, and usually associated with age-related disorders such as cardiac diseases [138]. In the case of elevated cell death and necrotic signalling, the IL33/STL2 pathway is triggered by Toll-Like Receptor-1 (TLR1) activation which leads to upregulation of the cytokine and chemokine influx [139]. A recent longitudinal study by Romero et al. reported significant elevation of sST2 levels in maternal plasma in risk patients almost 6 weeks prior to development of preeclampsia [140].

MicroRNAs (miRNA) and long noncoding RNAs (lncRNA) are noncoding RNAs that have the potential for gene regulation [141]. miRNAs are available in bodily fluids and are now being regularly investigated as both biomarkers and perspective therapeutics. In the uterus, miRNAs are controlled by steroidal hormones and they are responsible for efficient placentation. Recent work by Tan et al. stated that in adverse placental conditions like preeclampsia, long noncoding RNA, lncRNA DLX6-AS1, is overexpressed in trophoblastic cell lines, resulting in upregulation of mir-376c which is responsible for cell cycle arrest [142].

## 7. Potential Therapeutics and Future Perspectives for Adverse Pregnancy Outcomes

Ageing is a unidirectional process which cannot be reversed; however, research focused on reducing oxidative stress by restoring mitochondrial fitness could be a potential therapy for adverse pregnancy outcomes [143]. Mitochondrial fitness is acknowledged as the key regulator for cell cycle progression and metabolism with mitochondrial interactions with other organelles including the nucleus and endoplasmic reticulum (ER) ensuring homeostasis of cellular dynamics and viability. In pregnancy complications, chronic oxidative stress leads to mitochondrial dysfunction resulting in loss of mitochondrial signalling stability [102]. Antioxidant supplements such as vitamins C and E given during pregnancy have not demonstrated any significant effects in reducing oxidative stress.

However, continued research for more effective antioxidants have shed light on a few cytoprotective agents targeting the mitochondria.

A recent study has demonstrated that a mitochondria targeting antioxidant, MitoTEMPO, is cytoprotective against ROS-induced cell death [134]. When HUVEC cells were pretreated with MitoTEMPO before exposure to 3% plasma from preeclampsia pregnancies, it resulted in significant reduction in cellular superoxide production, normalization of mitochondrial functioning, and reduction in inflammatory influx [134]. Recently, another study done by Nuzzo et al. demonstrates that mitochondrial-targeting antioxidant MitoQ-treated pregnant rats exhibit normalized placental volumes and rescued fetal growth restriction in hypoxic conditions [144]. Furthermore, they discussed that MitoQ has the potential to regulate the cell cycle through the MAPK proliferation signalling pathway [144].

Finally, ergothioneine is a water soluble amino acid that has been shown to be cytoprotective. Ergothioneine primarily targets the mitochondria and can scavenge ROS, induce cellular proliferation, and repair through upregulated antioxidant pathways [145]. The multitude of benefits of antioxidants targeting and normalizing mitochondrial functioning and dynamics may become potential prenatal therapeutics for adverse pregnancy outcomes [145].

## 8. Conclusion

Senescence is beneficial to multicellular organisms especially for elimination of mutations and prevention of cancers. However, in the presence of persistent undesirable stress, this process can accelerate unnaturally and result in serious health complications. In adverse pregnancy outcomes such as preeclampsia and fetal growth restriction, placental insufficiency is accompanied with aberrant signalling for premature placental senescence and apoptosis. As summarized through Figure 1, this review has highlighted the current knowledge on the role of placental mitochondrial dysfunction, cellular senescence, and the placental ageing axis in adverse pregnancy outcomes.

Future research should focus on furthering our understanding of premature placental ageing as a pathophysiology in pregnancy disorders and investigating novel ways to improve outcomes such as preeclampsia and fetal growth restriction.

## Figures and Tables

**Table 1:** Senescence markers associated with placental ageing in adverse pregnancy outcomes.

Maternal plasma/serum	(1) Mitochondrial dysfunction (2) Inflammatory marker (IL33/ST2 signalling pathway)	↑ mtDNA copy numbers ↑ sST2 level

Trophoblastic cell lines	(1) Cell cycle regulators (2) miRNA/lncRNA (3) DNA damage response (4) Senescence regulators (5) Epigenetic changes	↑ Cyclin D1 ↑ Mir-376 ↑ PARP1 ↑ p53 ↓ JunB ↓ MDM2 ↑Hypomethylation of cytosine

Placenta	(1) DNA damage response (2) Cell cycle regulators (3) Mitochondrial dysfunction	↑ p53 ↓ hTERT ↑ Telomere aggregates ↑ BAX ↑Lipid peroxidation ↑ NOS activity ↑ Peroxynitrite synthesis ↑ Phosphorylated DERP1 gene
